# How Does a Robotic Lung Resection Programme Evolve? Learning Curve and Conversion Analysis of the Versius CMR Surgical System

**DOI:** 10.3390/cancers18162554

**Published:** 2026-08-09

**Authors:** Lubna Bakr, Hanna Binti Mohd Azhari, Muhammad Saad Asif, Mandip Chaubey, Adam Peryt, Aman S. Coonar, Giuseppe Aresu

**Affiliations:** Department of Cardiothoracic Surgery, Royal Papworth Hospital NHS Foundation Trust, Cambridge CB2 0AY, UK

**Keywords:** robotic-assisted thoracic surgery, learning curve, CUSUM, lung resection, conversion, Versius

## Abstract

**Background/Objectives**: Robotic-assisted thoracic surgery continues to spread internationally for anatomical lung resection, yet evidence on learning curve dynamics and conversion behaviour remains limited beyond the da Vinci system. The published clinical experience on the Versius CMR Surgical System does not yet match that of the da Vinci system. This study examines the learning curve and conversion pattern of a mature Versius programme using cumulative sum (CUSUM) analysis. **Methods**: Single-centre retrospective cohort study encompassing 206 consecutive anatomical lung resections (154 lobectomies, 52 segmentectomies) undertaken using the Versius CMR platform between April 2023 and February 2026. CUSUM analysis was applied at programme and individual surgeon level. Logistic regression was performed to identify independent predictors of conversion. Operative time and post-operative length of stay were examined, along with a comparative sub-analysis between converted and pure robotic cases, stratified by surgeon. **Results**: The programme-level CUSUM inflection point occurred at case 49 (7.3 months), with mean operative time falling from 250.9 ± 65.4 min in the early phase to 215.5 ± 46.8 min in the late phase (*p* = 0.001). Post-operative LOS improved from 5.8 to 4.7 days (*p* = 0.031). Forty-six cases (22.3%) required conversion, 87% of which were elective, with no significant change across programme phases (*p* = 1.00). A six-fold difference in conversion rate existed between the two main surgeons (35.4% vs. 6.1%). Surgeon-1’s conversions carried no operative time penalty (225.2 vs. 223.3 min, *p* = 0.96) or LOS penalty (3.9 vs. 5.0 days, *p* = 0.83), whereas Surgeon-2’s converted cases were significantly longer (333.3 vs. 216.9 min, *p* = 0.004) and associated with longer hospital stay (8.8 vs. 4.7 days, *p* = 0.004). On multivariable analysis, surgeon identity (aOR 5.39, 95% CI 2.25–12.92, *p* < 0.001), intra-operative adhesions (aOR 3.41, 95% CI 1.53–7.64, *p* = 0.003) and clinical stage II or higher (aOR 3.55, 95% CI 1.43–8.79, *p* = 0.006) were each independently associated with conversion. **Conclusions**: The Versius CMR system supports a definable and reproducible learning curve in anatomical lung resection. Conversion was determined both by operative difficulty and by individual surgeon decision-making philosophy, the latter accounting for the greater share of the programme’s conversion burden. Early elective conversion, when indicated, preserves operative efficiency and post-operative recovery, and should be viewed as wise surgical judgement, not a marker of programme immaturity.

## Simple Summary

Robotic surgery for lung resection is expanding worldwide. Versius is a robotic system that has been recently introduced to thoracic surgery. It has not been as thoroughly studied as more established systems. The surgeon may change the approach during a robotic operation to a traditional keyhole or open approach; this is known as conversion. We studied 206 consecutive lung operations undertaken using the Versius robot over almost 3 years to better understand the development of surgeons’ skills and what influenced their decision to convert. Surgical teams have become more efficient, and patients have recovered sooner as experience matured. Conversion depended more on each surgeon’s decision-making style and cautious approach. Choosing to convert early, when appropriate, protected patients rather than putting them at risk.

## 1. Introduction

Minimally invasive approaches have transformed the surgical management of lung cancer over the past few decades. Benefits of video-assisted thoracic surgery (VATS) have been demonstrated, establishing the VATS approach as the superior technique over thoracotomy. VATS achieved reduced postoperative complications, shorter hospital stay, and improved patient outcomes [1,2]. The uniportal VATS approach has offered further refinement of the approach with satisfactory feasibility and safety in selected centres with high-volume experience [3].

Robotic-assisted thoracic surgery (RATS) represents a further step in the evolution of minimally invasive lung resection. RATS offers satisfactory results and addresses technical limitations inherent to conventional VATS. It offers three-dimensional optics, instrument articulation beyond the degrees of freedom of the human wrist, and the elimination of physiological tremor [4,5]. Current evidence indicates broadly comparable perioperative outcomes between RATS and VATS in major lung resection [6,7,8,9,10], with any advantage appearing procedure- and centre-dependent.

The overwhelming majority of published RATS experience in the literature comes from programmes using the da Vinci Surgical System (Intuitive Surgical, Sunnyvale, California) [4,7,11,12]. The Versius Surgical System (CMR Surgical Ltd., Cambridge, UK) represents a newer robotic platform designed with a portable architecture and independently manoeuvrable bedside units, enabling flexible configuration of the operating room [13,14]. Preclinical evaluation of Versius in thoracic surgery demonstrated its feasibility in cadaveric models [15]. Fra et al. reported the first clinical evaluation of its feasibility and performance in thoracic surgery, looking into a small cohort of 30 cases including thymectomy and lung resection [16]. Most recently, our group published the first United Kingdom and second worldwide study of the Versius system in thoracic surgery, demonstrating safe implementation across 124 patients, including both anatomical lung resections and thymectomies, along with a propensity score-matched analysis demonstrating outcomes comparable to uniportal VATS [17].

A critical and often underreported dimension of any new robotic programme is the learning curve. This encompasses the safety profile, progressive improvement in operative efficiency, and confidence gained following adoption of a new robotic platform. The learning curve of RATS in lung resection has been reported primarily for the da Vinci platform, with published inflection points ranging from 20 to 150 cases [11,18,19,20]. Cumulative sum (CUSUM) analysis allows determination of the inflection point at which performance shifts from active skill acquisition to consistent reproducible performance [21,22]. To date, no CUSUM-based learning curve analysis has been performed for the Versius system in thoracic surgery.

Conversion from RATS to VATS or open thoracotomy is a significant and clinically relevant outcome in any new robotic programme. Whilst conversion has traditionally been looked at as a complication or failure, evidence suggests that elective, safety-driven conversion reflects sound surgical judgement, and that conversion rates decline with programme maturity [20,23]. The relationship between learning curve progression and conversion rate is rarely studied and is not described for Versius. In our initial publication, the learning curve was demonstrated as a simple moving average of operating times; however, that encompassed both thymectomy and lung resection cases and did not apply CUSUM methodology or examine conversion in detail [17]. Currently, with a substantially larger and more mature lung resection dataset, we are better positioned to generate a more rigorous characterisation of learning curve and conversion trends.

This study aims to characterise the learning curve of a mature Versius robotic-assisted programme for anatomical lung resection using CUSUM analysis at both programme and individual surgeon levels. It also aims to evaluate the conversion from RATS and examine how this evolves along with programme maturity.

## 2. Materials and Methods

### 2.1. Ethical Statement

The present study was approved by the Institutional Review Meeting and assigned the registration number STA-172.

### 2.2. Study Design and Patient Population

This is a single-centre retrospective cohort study conducted at the Department of Cardiothoracic Surgery, Royal Papworth Hospital NHS Foundation Trust, Cambridge, United Kingdom. It encompasses all consecutive patients who underwent robotic-assisted anatomical lung resection, either lobectomy or segmentectomy, using the CMR Versius system. It spans the period starting with its clinical implementation in April 2023 until February 2026. Patients undergoing different types of surgery using the Versius platform were excluded to allow a focused analysis of the anatomical lung resection learning curve. Surgeries were performed by 3 lead surgeons without prior robotic experience.

Cases were not allocated between surgeons according to complexity. As a matter of routine scheduling, the first case on each operating list was undertaken using the robotic platform, and no patient-, tumour- or physiology-based criteria governed selection for a robotic rather than a VATS approach. Complex sleeve resections or pneumonectomies were undertaken by uniportal video-assisted thoracoscopic surgery or open thoracotomy throughout the study period and are therefore not represented in this cohort.

### 2.3. Surgical Team and Programme Structure

The robotic programme at our institution involves three consultant thoracic surgeons, each with high-volume uniportal VATS experience (>150 cases per annum) but no prior robotic experience before implementation of Versius in April 2023 [17]. Bedside surgeons, fellows or residents, participated in each case at the bedside, and were responsible for manipulation of robotic arms, stapling, and completion of manual tasks under direct instruction from the console surgeon [17]. All theatre staff completed dry-laboratory training prior to participation in clinical robotic lists [17]. The scrub team was not fixed across the study period, reflecting real-world programme conditions [17].

### 2.4. Operative Technique

Anatomical lung resections were undertaken using 3 instrument bedside units and 1 visualisation bedside unit, with CO2 insufflation [17]. A single bedside assistant was present throughout, with one utility port [17]. The port configuration did not change materially over the study period. Further technical details have been described in our previous study [17].

The decision to convert was made by the console surgeon in discussion with the bedside surgeon and anaesthetist, taking account of the patient’s physiological status, the wish to avoid prolonged anaesthesia, and the operating list that day. Where the difficulty could be addressed thoracoscopically, the procedure was completed by uniportal VATS; conversion directly to thoracotomy was reserved for problems requiring immediate open access.

### 2.5. Statistical Analysis

Continuous variables were reported as mean and standard deviation, whereas categorical variables were reported as count and percentage. Statistical significance was defined as *p* < 0.05. The primary aspect for learning curve analysis was operative time. CUSUM analysis was performed as the principal methodology for examining learning curve phases and identifying inflection points and shifts in surgical performance. The CUSUM was constructed by subtracting a fixed reference value from each case’s operative time and adding these differences cumulatively, in chronological order (CUSUM = previous CUSUM + [operative time − 224 min], beginning at zero before the first case). The reference value was the arithmetic mean operative time of the series. A case longer than the reference contributes a positive value, and the curve rises; a case shorter than the reference contributes a negative value, and the curve falls. The curve therefore ascends while cases are running slower than the series average and descends once they are consistently running faster. No risk adjustment, weighting or predefined decision limits were applied, as the analysis was intended to characterise the learning curve descriptively rather than to monitor performance against an acceptable failure rate.

The inflection point was defined a priori as the case number at which the CUSUM curve reached its maximum value, rather than by visual inspection of the curve. Cases up to and including this point were designated the early phase, and subsequent cases the late phase. For per-surgeon analyses, each surgeon’s own mean operative time was used as the reference value; both principal surgeons had a mean operative time of 224 min, identical to the programme mean, and the inflection points were therefore unchanged whether the individual or programme reference was applied.

Because different CUSUM formulations may yield different thresholds, the inflection point was tested against alternative specifications: substituting the median (220 min) or a 5% trimmed mean for the reference value, and repeating the analysis on log-transformed operative time. All specifications identified the same inflection point. Operative time was unavailable for one patient; therefore, 205 of 206 cases contributed to the CUSUM analysis.

CUSUM analysis was performed at the institution level, and at individual surgeon level. Individual CUSUM curves were constructed only for the two surgeons with sufficient caseload. Secondary learning curve metrics included a moving average of operating time plotted by consecutive calendar months. Statistical analysis was performed using Python (version 3.12). Mann–Whitney U test was used for comparisons of continuous variables between two groups, and Kruskal–Wallis test for comparisons across three or more groups. Chi-square test was used for categorical comparisons. Spearman rank correlation was used to assess the association between continuous variables. Logistic regression was performed to identify independent predictors of conversion, with results reported as odds ratios (OR) and adjusted odds ratios (aOR) with 95% confidence intervals. Right lower lobe (RLL) was used as the reference lobe category in the regression model. Bi-lobectomies were excluded from models containing the lobe variable. A sensitivity analysis using the right upper lobe (RUL) as reference was also performed and yielded consistent results with no change in clinical interpretation. Crude odds ratios (OR) from univariable analysis are reported alongside adjusted odds ratios (aOR) from multivariable analysis to allow assessment of the independent effect of each predictor after controlling for potential confounders. Model performance was assessed by discrimination (area under the receiver operating characteristic curve) and calibration (calibration slope and Hosmer–Lemeshow goodness-of-fit test). Optimism arising from model complexity was estimated by bootstrap resampling with 400 replicates, and optimism-corrected estimates are reported alongside apparent values. No stepwise or data-driven variable selection was performed; predictors were prespecified on clinical grounds.

One case was excluded from the post-operative length of hospital stay (LOS) comparison of converted cases only, as the robotic approach could not be established due to inability to manoeuvre the robotic arms in a patient with a BMI of 42, and the procedure was completed entirely by VATS. Its LOS therefore reflects that of a primary VATS procedure rather than a robotic conversion. This case is included in all other analyses.

Operative time includes robot setup, docking and undocking; anaesthetic time is excluded. The presence and severity of intra-operative adhesions were retrieved retrospectively from the operative record for all patients. Lymphadenectomy was recorded as the number of lymph nodal stations dissected, in keeping with institutional histopathology reporting practice. Conversions were classified as emergency where active haemorrhage necessitated urgent conversion, and as elective otherwise.

Propensity matching was not applied, as the analysis was single-arm and concerned temporal maturation within one cohort rather than comparison between treatment groups.

## 3. Results

### 3.1. Patient Cohort

A total of 206 patients underwent robotic-assisted anatomical lung resection using the Versius CMR Surgical System between April 2023 and February 2026. Of these, 154 underwent lobectomy, and 52 underwent anatomical segmentectomy. Baseline characteristics are summarised in Table 1. Mean age was 69.6 ± 8.9 years, and 40.3% (83/206) were male. Mean FEV_1_% predicted was 88.5 ± 19.5%, and mean DLCO 82.8 ± 19.0%. The most frequently resected lobe was the right upper lobe (RUL) (35.4%).

### 3.2. Learning Curve Analysis

**Figure 1:**
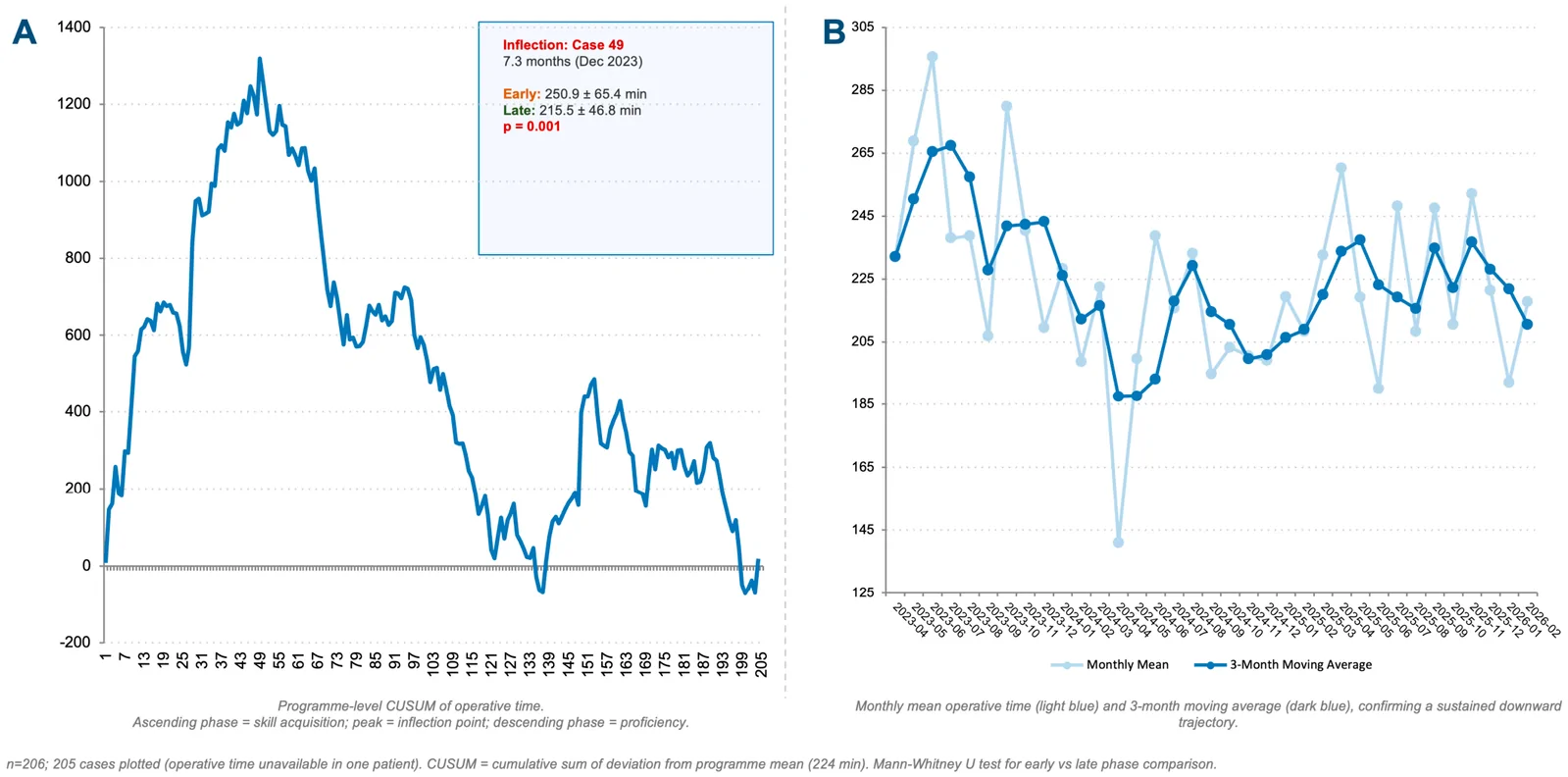
Programme-Level Learning Curve—CUSUM Analysis (**A**) and Monthly Operative Time Trend (**B**).

The programme-level CUSUM curve for operative time is shown in Figure 1A. An initial ascending phase reflecting active skill acquisition was observed, followed by an inflection point at case 49, occurring approximately 7.3 months following programme initiation. Mean operative time in the early phase (cases 1–49) was 250.9 ± 65.4 min, compared with 215.5 ± 46.8 min in the late phase (cases 50–205) (*p* = 0.001), representing an overall reduction of 35.4 min (14.1%).

**Figure 2:**
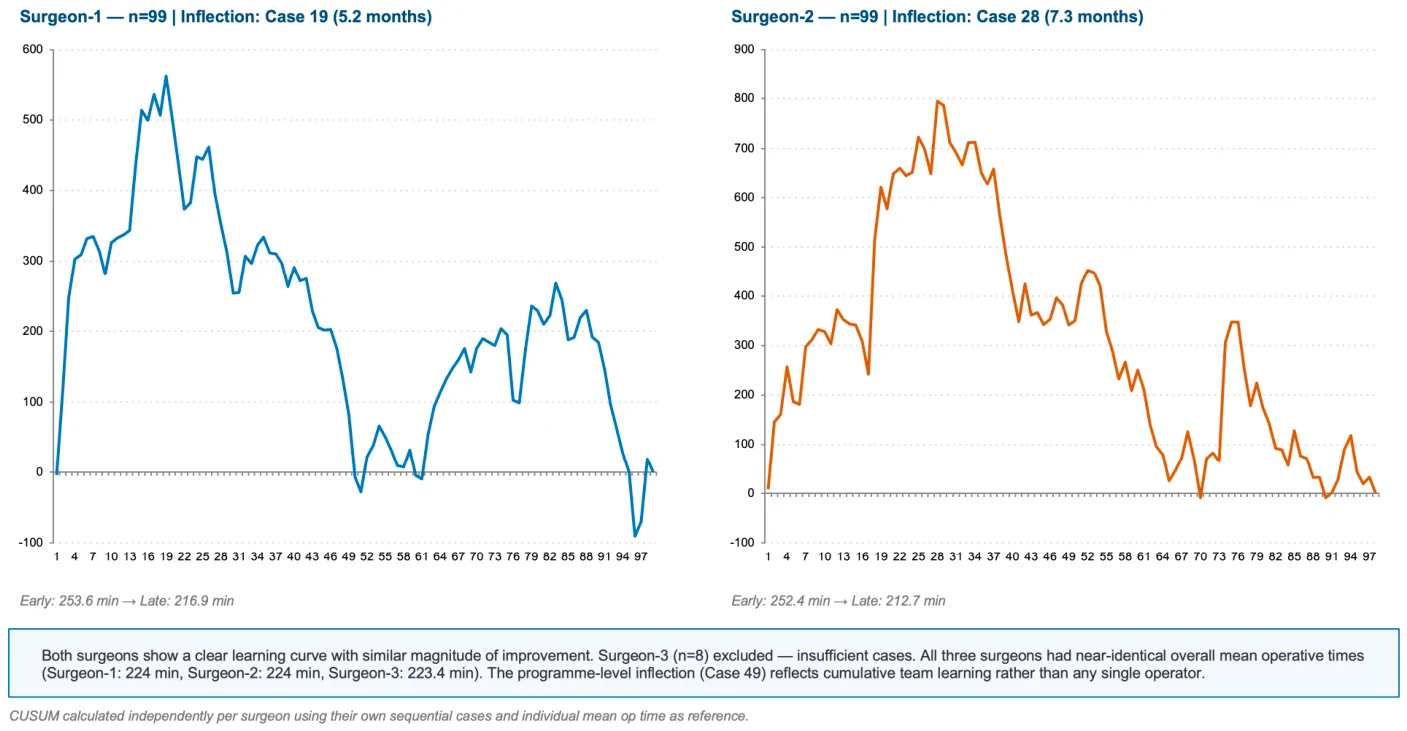
Per-Surgeon CUSUM—Operative Time.

At individual surgeon level (Figure 2), CUSUM analysis identified inflection points at case 19 for Surgeon-1 and case 28 for Surgeon-2, while Surgeon-3 performed only eight cases during the study period, which was insufficient for meaningful CUSUM analysis. Moving average analysis by calendar month confirmed a consistent downward trajectory in operative time, from a mean of 250.9 min in the early phase to a late-phase mean of 215.5 min (*p* = 0.001) (Figure 1B). Baseline characteristics did not differ significantly between the early and late programme phases (all *p* > 0.05), confirming that improvement in operative time reflected learning rather than patient selection.

Post-operative length of stay (LOS) also improved significantly between programme phases, from a mean of 5.8 days in the early phase to 4.7 days in the late phase (*p* = 0.031), providing a secondary indicator of programme maturation. Longer operative time was associated with longer post-operative hospital stay (Spearman rho 0.198, *p* = 0.004); mean stay was 6.9 days in the longest operative-time quartile compared with 3.8 days in the shortest (*p* = 0.014).

### 3.3. Conversion Analysis

**Figure 3:**
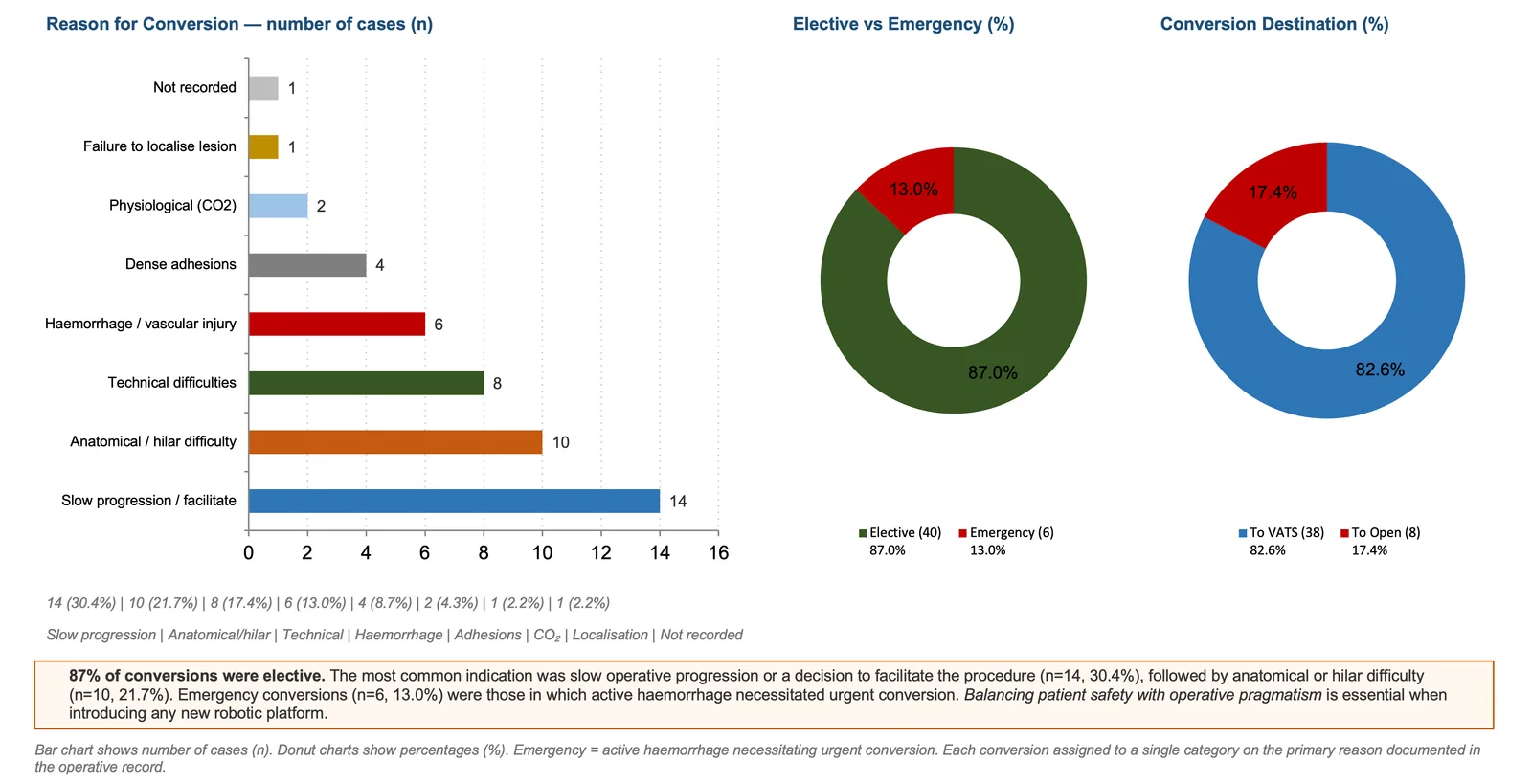
Conversion—Reasons (n), Destination (%), and Elective Status (%).

Forty-six (22.3%) patients required conversion from the robotic approach. Of these, 38 (82.6%) were converted to uniportal VATS, and eight (17.4%) to open thoracotomy, the latter representing 3.9% of the whole cohort. The most common indication was slow operative progression or a decision to facilitate the procedure (n = 14, 30.4%), followed by anatomical or hilar difficulty (n = 10, 21.7%), technical difficulties (n = 8, 17.4%), haemorrhage or vascular injury (n = 6, 13%), dense adhesions (n = 4, 8.7%), physiological intolerance of carbon dioxide insufflation (n = 2, 4.3%) and failure to localise the lesion after frozen section, converted for better palpation (n = 1, 2.2%). No indication was recorded in one case. The decision to convert was made electively in 87% of cases (Figure 3).

The single 30-day death occurred in a converted case. Conversion from robotic to thoracoscopic and then open surgery was prompted by instrument clashing in a small chest, with an anatomical variation requiring reanastomosis of a pulmonary vein. The patient required post-operative extracorporeal membrane oxygenation and died of multi-organ failure on the second post-operative day.

Conversion rate did not differ significantly between the early and late programme phases (22.4% vs. 22.3%, *p* = 1.00). A marked difference in conversion rate was observed between the two main operating surgeons: Surgeon-1 converted 35/99 cases (35.4%), compared with 6/99 (6.1%) for Surgeon-2, representing a six-fold difference. This surgeon-level variation was the dominant driver of the overall programme conversion rate. Within each surgeon’s own caseload, conversion rate fell across successive thirds: Surgeon-1 from 45% to 39% to 21% (*p* = 0.10) and Surgeon-2 from 9% to 6% to 3% (*p* = 0.59), neither significantly. The stable programme-level rate reflects an increase in Surgeon-1’s share of the caseload, from 39% of early-phase cases to 51% of late-phase cases.

**Figure 4:**
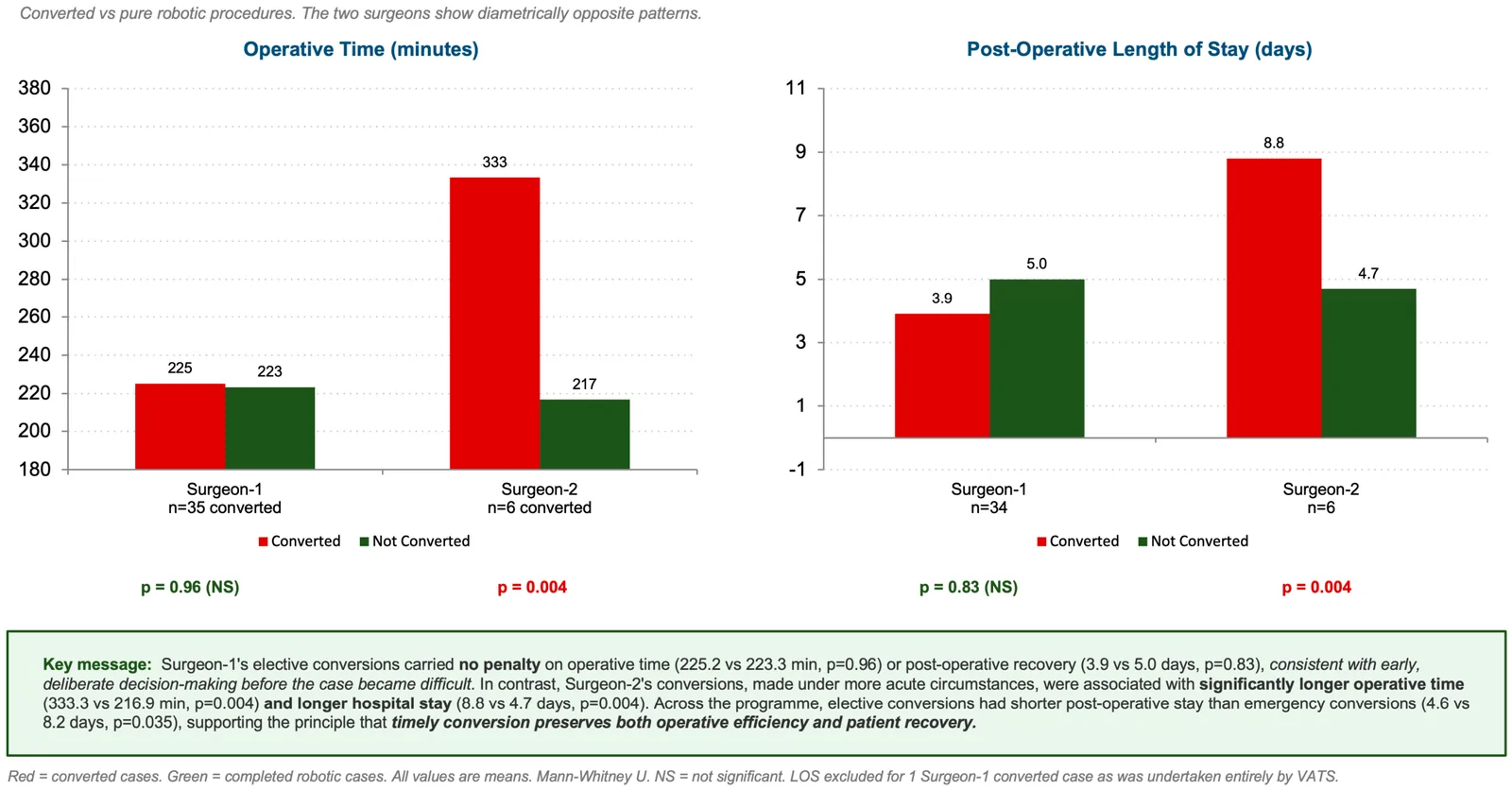
Impact of Conversion on Operative Time and Post-Operative LOS—by Surgeon.

When total operative time was compared between converted and non-converted cases for each surgeon independently, a clear difference in conversion behaviour emerged (Figure 4). For Surgeon-1, total operative time was virtually identical whether a case was converted or not (225.2 vs. 223.3 min, *p* = 0.96), suggesting that conversions were made early and electively in the procedure, before significant additional time had elapsed. In contrast, for Surgeon-2, converted cases were dramatically longer than pure robotic cases (333.3 vs. 216.9 min, *p* = 0.004), consistent with conversion under more acute or demanding circumstances. These findings indicate two distinct conversion philosophies. Surgeon-1 applied a low and consistent threshold for elective conversion, whilst Surgeon-2 persisted robotically in nearly all cases and converted only when absolutely necessary.

On multivariable logistic regression, three factors were independently associated with conversion: Surgeon-1 as operator (aOR 5.39, 95% CI 2.25–12.92, *p* = 0.0002), the presence of intra-operative adhesions (aOR 3.41, 95% CI 1.53–7.64, *p* = 0.003) and clinical stage II or higher (aOR 3.55, 95% CI 1.43–8.79, *p* = 0.006). Programme phase, resection extent and target lobe were not independently associated with conversion (Table 2). Because eight predictors and 44 conversion events yielded fewer than 10 events per variable [24], a reduced four-variable model was also fitted, in which surgeon identity remained independently associated with conversion (aOR 4.63, 95% CI 2.04–10.50, *p* = 0.0002). Bi-lobectomies were excluded from models containing the lobe variable. The full model achieved an apparent area under the receiver operating characteristic curve of 0.795, falling to 0.752 after bootstrap correction for optimism, with a corrected calibration slope of 0.792. The reduced four-variable model showed less optimism and better corrected performance (apparent AUC 0.780, corrected 0.761; calibration slope 0.909). Neither model showed evidence of poor fit (Hosmer–Lemeshow *p* = 0.835 and *p* = 0.981, respectively).

Case characteristics were compared between the two principal operating surgeons across demographic, physiological, oncological and operative variables (Table 3). Adhesions were present in 54 of 206 patients (26.2%) and were strongly associated with conversion (15.8% in patients without adhesions versus 40.7% in those with, *p* < 0.001). Adhesion burden did not differ between the two principal surgeons (28.3% versus 25.3%, *p* = 0.75). Within patients who had no adhesions, Surgeon-1 converted 19 of 71 cases (26.8%) and Surgeon-2, 1 of 74 (1.4%, *p* < 0.001); among those with adhesions, the rates were 16 of 28 (57.1%) and 5 of 25 (20.0%, *p* = 0.011). The difference between surgeons therefore persisted within each stratum of operative difficulty.

When converted cases were excluded from operative time analysis, no significant difference in operative efficiency was observed between Surgeon-1 and Surgeon-2 for pure robotic procedures (223.3 vs. 216.9 min, *p* = 0.16), further supporting that the conversion rate difference reflects decision-making threshold rather than operative speed.

There was one 30-day mortality in the series, occurring in a converted case (1/46, 2.2%). No mortalities occurred among completed robotic cases.

Converted and non-converted cases did not differ significantly in post-operative LOS (5.1 vs. 4.7 days, *p* = 0.45).

### 3.4. Impact of Conversion on Operative Time and Post-Operative Length of Stay

Surgeon-2’s converted cases were associated with a significantly longer post-operative stay compared with his pure robotic procedures (mean 8.8 vs. 4.7 days, *p* = 0.004), in contrast to Surgeon-1’s converted cases, which carried no such penalty (mean 3.9 vs. 5.0 days, *p* = 0.83). Across the programme, elective conversions had a shorter post-operative stay than emergency conversions (mean 4.6 vs. 8.2 days, *p* = 0.035), consistent with the findings of Servais et al. [25]. These findings suggest that a policy of early elective conversion, when the robotic approach is not progressing optimally, may preserve operative efficiency and protect post-operative recovery.

## 4. Discussion

This study presents the first CUSUM-based learning curve analysis for the Versius CMR Surgical System in thoracic surgery, and is the first to examine the evolution of conversion rate as a function of programme maturity for this platform. The programme reached a definable inflection point at 49 cases and 7.3 months. On comparison with the da Vinci-based programmes, it is higher than the 18–32 cases reported in single-surgeon series [18,20], but substantially lower than the institution-level plateau of 150 cases reported by Walker et al. [19]. Conversion rate remained stable throughout the study period, and the majority of conversions were made electively, reflecting safety-driven decision-making. The most striking finding was the markedly different conversion philosophy between the two main operating surgeons, which had important implications for how overall programme conversion rates should be interpreted.

### 4.1. Learning Curve

CUSUM analysis is the accepted standard for objective assessment of surgical learning curve, as it is sensitive to performance shifts without requiring a pre-specified threshold [21,22]. Its application to robotic thoracic surgery learning curves remains limited, with most published analyses relying on moving average or subjective time-trend plots [26]. Our study applies CUSUM at both programme and individual surgeon level, providing the first such analysis for the Versius CMR Surgical System in thoracic surgery.

Published inflection points for RATS lobectomy using the da Vinci system range from 20 to 150 cases, depending on the analytical method, outcome measure, and prior experience of the surgeon [11,19,20]. Veronesi et al. reported improvement in operating times and median hospitalisation after approximately 18 cases in a single-surgeon series using the da Vinci system [20]. Gómez-Hernández et al. also evaluated a single-surgeon series, using CUSUM analysis, and identified an inflection point at case 32 transitioning from VATS to robotic anatomical lung resection [18]. This is the most comparable setting to ours, given the extensive prior uniportal VATS experience of our operating surgeons. Walker et al., in a large population-based study of institution-level robotic learning curves across surgical specialties, reported a range of 75–300 cases before reaching stable performance [19]. This analysis was not restricted to thoracic surgery; however, it reported reaching plateaus after 150 cases of robotic lobectomy [19]. Our programme-level inflection point of 49 cases sits well between the single-surgeon series [18,20] and the institution-level analyses [19], which would be expected from a multi-surgeon programme. It reflects the cumulative performance of the three lead surgeons performing cases sequentially, rather than the individual trajectory of a single operator. As such, it represents a measure of team-level learning rather than individual proficiency, and should be interpreted accordingly as an indicator of institutional programme maturity.

It should also be noted that the learning curve in robotic surgery, especially with Versius, extends well beyond the console surgeon, as stapling remains a step dependent on the bedside surgeon throughout the operation, given that integrated robotic stapling is not yet available on this platform [17]. The bedside surgeon’s proficiency in anticipating and facilitating stapler deployment, as well as their familiarity with the operating style and instructions of the console surgeon, directly influences operative time and procedural flow. As such, our programme-level CUSUM curve reflects team learning as much as individual surgical proficiency, and this should be considered when benchmarking against single-surgeon da Vinci series in the literature.

The earlier publication from our group reported a learning curve inflection at approximately 66 cases and 7 months, using moving average analysis across both lung resections and thymectomies [17]. The current analysis applies CUSUM methodology on a larger number of pure anatomical lung resection cases, providing a more rigorous characterisation of learning trajectory. The per-surgeon analysis additionally reveals whether learning was distributed evenly across the two operating surgeons, which has practical implications for programme planning in centres anticipating multiple robotic operators.

Operative times in this series exceeded those typically reported for contemporary VATS anatomical resection, and longer operations were associated with longer post-operative stay. Reducing operative duration is therefore not merely a measure of efficiency, and this consideration supports a low threshold for conversion when robotic progress is slow.

Our own propensity score-matched comparison found Versius outcomes comparable rather than superior to uniportal VATS [17], and the current platform lacks integrated stapling and yields longer operative times than our thoracoscopic practice. The present findings should be interpreted in the context of the operating team’s prior experience. Each surgeon had performed more than 150 uniportal VATS anatomical resections annually before robotic implementation. Centres without an established high-volume thoracoscopic practice should anticipate a longer learning curve. The curve also reflects team rather than individual proficiency, since stapling on the Versius platform is undertaken by the bedside surgeon; units with a fixed robotic team may therefore progress differently from those with rotating theatre staff.

### 4.2. Conversion

Conversion rates in published RATS lobectomy studies reported a rate of 0% in uniportal RATS study by Paradela et al., and 0.7% by Park et al. [7,23]. Both papers focused on conversion to thoracotomy without clarifying whether conversion to VATS was necessary [7,23]. In our initial publication, we reported an overall RATS conversion rate of 22.58% across lung resections and thymectomies, and highlighted that the majority were performed electively [17]. The current analysis of a larger and more mature pure lung resection dataset demonstrates that the overall conversion rate remained stable at 22.3%, with no statistically significant reduction between the early and late programme learning phases (22.4% vs. 22.3%, *p* = 1.00). This contrasts with the expected trajectory described in some da Vinci series, and therefore should be carefully interpreted. Our figure counts any departure from the robotic approach, whereas contemporary series generally report conversion to thoracotomy alone; on that definition, our rate was 3.9% (8 of 206). Conversion rates are therefore not comparable between series unless the definition is stated. This is reflected in pooled analyses, where robotic conversion rates are lower than thoracoscopic overall (OR 0.53, 95% CI 0.35–0.83) but with substantial heterogeneity between series (I^2^ = 82%) [27].

Cerfolio et al., in their consecutive experience with robotic lobectomy using the da Vinci system, described a reduction in conversion rate from 19% in early cases to 1% as the programme matured [28]. They reported that elective conversion was decided to a time limit of 4 h to prevent patient injury from prolonged anaesthesia and to limit both operating room personnel and surgeon frustration [28]. In our series, the conversions undertaken for slow progression had a mean operative time of 216 min, so a four-hour limit would rarely have been the operative trigger; a low and consistently applied threshold appears more relevant than an absolute time limit. Our initial study reported that elective conversion was decided in 25% of the conversions to facilitate the progression of the procedure and keep the operating time to a minimum [17]. Now, however, with a more mature programme and a larger dataset, we note that the conversion rate has been stable across phases. One platform difference is relevant here; stapling is integrated on the da Vinci system but is undertaken by the bedside surgeon on the Versius platform, so control of the critical step and the operative flow around it differ. The findings highlight an important difference between surgeons. Surgeon-1 converted 35.4% of cases electively and early, with total operative times virtually identical to those of his pure robotic cases (225.2 vs. 223.3 min, *p* = 0.96). Surgeon-2 maintained a 6.1% conversion rate, converting only under more acute circumstances, with operative times for converted cases being significantly longer than his pure robotic procedures (333.3 vs. 216.9 min, *p* = 0.004). These two patterns reflect fundamentally different conversion philosophies rather than different operative speeds; when only pure robotic cases are compared, no significant difference in operative time exists between the two surgeons (223.3 vs. 216.9 min, *p* = 0.16). This supports the rationale behind conversion to facilitate case progression. Surgeon-1’s decision was made early enough that the combined robotic and VATS operative time was not prolonged beyond that of a pure robotic procedure, reflecting deliberate and timely decision-making rather than a last resort.

Cao et al. showed that conversion rates were highest in earlier cases and declined after the initial learning period [11]. They reported a variable conversion rate among studies from 0% up to 19.2% [11]. This relationship is not consistent across minimally invasive platforms; in a national VATS database, greater surgeon experience was not associated with a lower conversion rate [29]. Servais et al. showed that elective conversion was associated with reduced mortality compared with emergency conversion (*p* < 0.001) [25]. Our findings add an important dimension to this. Within a single programme, conversion rate reflects both operative difficulty and individual surgeon decision-making philosophy. Intra-operative adhesions and clinical stage were each independently associated with conversion [30], yet the difference between surgeons persisted after adjustment for both and within strata of adhesion burden, indicating that decision-making threshold operates in addition to, rather than instead of, case complexity. This has implications for how conversion rates are interpreted when comparing programmes in the literature. A high elective conversion rate, where total operative time is not prolonged and outcomes are not compromised, should not be equated with poor robotic proficiency.

Elective conversion should not be viewed as a failure, but rather an indicator of sound surgical judgement [31]. Throughout our programme, the decision to convert was made predominantly to ensure a safe approach or to facilitate operative progression, rather than due to haemorrhage or life-threatening complication [17]. Balancing patient safety with operative pragmatism in this way is essential when introducing any new robotic platform [28]. Three practical measures follow from this experience: maintaining a consistent bedside team, since operative time on this platform reflects team as much as console proficiency; applying a low threshold for early conversion, which carried no penalty in operative time or length of hospital stay; and accepting that the learning curve is a cost of adoption to be planned for rather than avoided.

### 4.3. Study Limitations

This study has a number of limitations. It is a single-centre observational analysis, with a relatively small individual surgeon caseload, which limits the statistical power of per-surgeon CUSUM curves. The three lead operating surgeons had extensive prior VATS experience but no prior robotic experience, which may not be applicable to centres introducing RATS without a strong minimally invasive foundation.

Operative time was used as the primary learning curve metric, as it is the most objectively measurable outcome, consistently reported in the literature. The absence of a concurrent da Vinci comparator group limits platform-specific conclusions, and future prospective studies comparing Versius directly with established robotic systems in thoracic surgery are needed.

The per-surgeon conversion analysis should be interpreted with caution given the observational nature of the study and the relatively small numbers, particularly for Surgeon-2’s converted cases (n = 6). Additionally, the logistic regression model has limited power, with 44 conversion events across eight predictors in the full model. A reduced four-variable model respecting the conventional 10-events-per-variable threshold produced consistent estimates and better optimism-corrected performance, but the multivariable findings should nonetheless be regarded as hypothesis-generating rather than definitive.

## 5. Conclusions

This is the first CUSUM-based learning curve analysis for the Versius CMR Surgical System in robotic anatomical lung resection. A programme-level inflection point was definable at 49 cases and 7.3 months, with sustained improvement in operative time thereafter. Conversion rate did not decline significantly over time, but remained predominantly elective (87%), reflecting safety-driven decision-making throughout. The most novel finding of this study was the markedly different conversion behaviour between the two main operating surgeons. Surgeon-1 applied a low elective conversion threshold with no impact on total operative time or post-operative length of stay, whilst Surgeon-2 maintained a near-complete robotic completion rate and converted only under compelling circumstances, with converted cases associated with significantly longer operative times and hospital stay. Across the programme, elective conversions were associated with shorter post-operative stay than emergency conversions, supporting the principle that timely, safety-driven conversion preserves operative efficiency and protects patient recovery. The findings support the safe adoption of the Versius system in thoracic surgical centres, provided that programmes are structured with adequate team training, proctored initiation, and a willingness to convert when operative safety demands it. Future studies are needed to examine how the learning curve trajectory translates to peri-operative outcomes as the programme continues to mature.

## Figures and Tables

**Table 1:** Baseline characteristics of the study cohort, overall and by programme phase. Values are n (%) or mean ± SD unless otherwise stated. Early phase = cases 1–49; late phase = cases 50–206. Mann–Whitney U test for continuous variables; chi-square or Fisher’s exact test for categorical variables. Denominators differ where data were unavailable.

Variable	All (n = 206)	Early (n = 49)	Late (n = 157)	*p* -Value
**Demographics**				
Age (years), mean ± SD	69.6 ± 8.9	69.6 ± 8.6	69.6 ± 9.1	0.886
Male sex, n (%)	83 (40.3%)	23 (46.9%)	60 (38.2%)	0.358
Current smoker, n (%)	49 (23.8%)	—	—	
Ex-smoker, n (%)	109 (52.9%)	—	—	
Never smoked, n (%)	45 (21.8%)	—	—	
FEV_1_% predicted, mean ± SD	88.5 ± 19.5	83.6 ± 20.5	90.1 ± 19.0	0.088
DLCO% predicted, mean ± SD	82.8 ± 19.0	83.0 ± 17.7	82.8 ± 19.5	0.874
COPD, n (%)	59 (28.6%)	17 (34.7%)	42 (26.8%)	0.372
**Oncological Characteristics**				
Neoadjuvant therapy, n (%)	2 (1.0%)	0 (0%)	2 (1.3%)	1.000
Clinical stage II or higher, n (%)	41/192 (21.4%)	9/47 (19.1%)	32/145 (22.1%)	0.826
T3/T4, n (%)	16/190 (8.4%)	4/46 (8.7%)	12/144 (8.3%)	1.000
**Operative Details**				
Surgeon-1, n (%)	99 (48.1%)	19 (38.8%)	80 (51.0%)	0.322
Surgeon-2, n (%)	99 (48.1%)	28 (57.1%)	71 (45.2%)	
Surgeon-3, n (%)	8 (3.9%)	2 (4.1%)	6 (3.8%)	
Lobectomy, n (%)	154 (74.8%)	42 (85.7%)	112 (71.3%)	0.067
Segmentectomy, n (%)	52 (25.2%)	7 (14.3%)	45 (28.7%)	
Adhesions present, n (%)	54 (26.2%)	11 (22.4%)	43 (27.4%)	0.617
Adhesions significant or extensive, n (%)	14 (6.8%)	4 (8.2%)	10 (6.4%)	0.745
Lymph nodal stations dissected, mean ± SD	3.4 ± 1.2	3.3 ± 1.2	3.4 ± 1.2	0.835
**Target Lobe**				
Right upper lobe (RUL)	73 (35.4%)	15 (30.6%)	58 (36.9%)	0.767
Right lower lobe (RLL)	59 (28.6%)	13 (26.5%)	46 (29.3%)	
Left lower lobe (LLL)	42 (20.4%)	13 (26.5%)	29 (18.5%)	
Left upper lobe (LUL)	20 (9.7%)	5 (10.2%)	15 (9.6%)	
Right middle lobe (RML)	10 (4.9%)	3 (6.1%)	7 (4.5%)	
Bi-lobectomy	2 (1.0%)	0 (0%)	2 (1.3%)	
**Outcomes**				
**Operative time (min), mean ± SD**	**224.0 ± 53.9**	**250.9 ± 65.4**	**215.5 ± 46.8**	**0.001**
Post-op LOS (days), median (IQR)	3.0 (2.0–5.0)	—	—	
**Post-op LOS (days), mean ± SD**	**4.9 ± 6.0**	**5.8 ± 5.9**	**4.7 ± 6.0**	**0.031**
Conversion to VATS or open, n (%)	46 (22.3%)	11 (22.4%)	35 (22.3%)	1.000
30-day mortality, n (%)	1 (0.5%)	—	—	
Air leak > 5 days, n (%)	29 (14.1%)	—	—	* *

**Table 2:** Logistic regression—predictors of conversion. OR = crude odds ratio. aOR = adjusted odds ratio. Reference categories: Surgeon-2/3, no adhesions, clinical stage I, early phase, segmentectomy, right lower lobe. Lobe abbreviations: RUL = right upper lobe, RLL = right lower lobe, LLL = left lower lobe, LUL = left upper lobe, RML = right middle lobe. RML was excluded from the model owing to complete separation (no conversions), as were the two bi-lobectomies. Full model AUC 0.795. Variance inflation factors ranged from 1.06 to 1.41, indicating no material collinearity. A sensitivity analysis using the right upper lobe as reference yielded consistent results, with surgeon identity remaining independently associated with conversion.

**Univariable analysis—factors associated with conversion**				
**Variable**	**OR**	**95% CI**	***p*** **-value**	**Sig**
**Surgeon-1 (vs. Surgeon-2/3)**	**4.67**	**2.21–9.88**	**<0.001**	**Yes**
**Intra-op adhesions present**	**3.61**	**1.80–7.24**	**<0.001**	**Yes**
**Clinical stage II or higher**	**3.70**	**1.75–7.82**	**<0.001**	**Yes**
Late phase (vs. Early)	1.01	0.47–2.17	0.985	No
Lobectomy (vs. Segmentectomy)	2.22	0.92–5.33	0.074	No
RUL (vs. RLL)	0.95	0.47–1.88	0.872	No
LLL (vs. RLL)	1.51	0.70–3.25	0.297	No
LUL (vs. RLL)	1.54	0.56–4.27	0.404	No
**Multivariable analysis—independent predictors of conversion**				
**Variable**	**aOR**	**95% CI**	***p*** **-value**	**Sig**
**Surgeon-1 (vs. Surgeon-2/3)**	**5.39**	**2.25–12.92**	**<0.001**	**Yes**
**Intra-op adhesions present**	**3.41**	**1.53–7.64**	**0.003**	**Yes**
**Clinical stage II or higher**	**3.55**	**1.43–8.79**	**0.006**	**Yes**
Late phase (vs. Early)	0.71	0.28–1.81	0.478	No
Lobectomy (vs. Segmentectomy)	1.45	0.44–4.82	0.541	No
RUL (vs. RLL)	2.40	0.86–6.67	0.093	No
LLL (vs. RLL)	1.84	0.62–5.41	0.270	No
LUL (vs. RLL)	3.00	0.75–12.02	0.120	No
**Reduced model—four predictors**				
**Variable**	**aOR**	**95% CI**	***p*** **-value**	**Sig**
**Surgeon-1 (vs. Surgeon-2/3)**	**4.63**	**2.04–10.50**	**<0.001**	**Yes**
**Intra-op adhesions present**	**3.62**	**1.65–7.95**	**0.001**	**Yes**
**Clinical stage II or higher**	**2.91**	**1.24–6.82**	**0.014**	**Yes**
Lobectomy (vs. Segmentectomy)	1.28	0.44–3.72	0.651	No

**Table 3:** Comparison of case characteristics between the two principal operating surgeons. Values are n (%) or mean ± SD. Mann–Whitney U test for continuous variables; chi-square or Fisher’s exact test for categorical variables. Denominators differ where data were unavailable. Adhesion burden, physiological reserve and oncological stage did not differ between surgeons.

Variable	Surgeon-1 (n = 99)	Surgeon-2 (n = 99)	*p*
**Demographics and physiology**			
Age, years	69.4 ± 9.6	70.1 ± 8.4	0.97
Male sex	41 (41.4%)	41 (41.4%)	1.00
FEV_1_ % predicted	88.1 ± 17.4	89.1 ± 21.1	0.95
DLCO % predicted	82.2 ± 19.2	83.6 ± 18.9	0.58
COPD	27 (27.3%)	30 (30.3%)	0.75
**Oncological characteristics**			
Neoadjuvant therapy	2 (2.0%)	0 (0%)	0.50
Clinical stage II or higher	23/92 (25.0%)	17/93 (18.3%)	0.35
T3/T4	9/90 (10.0%)	6/93 (6.5%)	0.55
T4	4/90 (4.4%)	0/93 (0%)	0.057
**Operative characteristics**			
Lobectomy (vs. segmentectomy)	80 (80.8%)	69 (69.7%)	0.10
**Lymph nodal stations dissected**	**3.1 ± 1.2**	**3.6 ± 1.1**	**0.003**
Adhesions present	28 (28.3%)	25 (25.3%)	0.75
Adhesions significant or extensive	10 (10.1%)	4 (4.0%)	0.16
**Target lobe**			
**Overall lobe distribution**	**—**	**—**	**0.002**
**Upper lobe resection (RUL/LUL)**	**36 (36.4%)**	**53 (53.5%)**	**0.022**
**Lower lobe resection (RLL/LLL)**	**61 (61.6%)**	**36 (36.4%)**	**<0.001**
**Outcome**			
**Conversion**	**35 (35.4%)**	**6 (6.1%)**	**<0.001**

## Data Availability

The data underlying this article will be shared on reasonable request to the corresponding author.

## Abbreviations

The following abbreviations are used in this manuscript:

VATS	Video-assisted thoracic surgery
RATS	Robotic-assisted thoracic surgery
CUSUM analysis	Cumulative sum analysis
OR	Odds ratio
aOR	Adjusted odds ratio
RLL	Right lower lobe
RUL	Right upper lobe
LOS	Length of hospital stay
RML	Right middle lobe
COPD	Chronic obstructive pulmonary disease
LLL	Left lower lobe
LUL	Left upper lobe
IQR	Interquartile range
SD	Standard deviation
